# The case for biotechnological exceptionalism

**DOI:** 10.1007/s11019-021-10032-5

**Published:** 2021-06-19

**Authors:** Jan-Hendrik Heinrichs

**Affiliations:** 1 Institut für Neurowissenschaften und Medizin, Forschungszentrum Jülich, Forschungszentrum Jülich, INM-8: Ethik in den Neurowissenschaften, 52425 Jülich, Germany; 2 Rheinisch-Westfälische Technische Hochschule (RWTH), Aachen, Germany

**Keywords:** Biomedical exceptionalism, Parity-principle, Enhancement, Informed consent

## Abstract

Do *biomedical* interventions raise special moral concerns? A rising number of prominent authors claim that at least in the case of biomedical enhancement they do not. Treating biomedical enhancements different from non-biomedical ones, they claim, amounts to unjustified biomedical exceptionalism. This article vindicates the familiar thesis that biomedical enhancement raises specific concerns. Taking a close look at the argumentative strategy against biomedical exceptionalism and provides counterexamples showing that the biomedical mode of interventions raises concerns not relevant otherwise. In particular, biomedical interventions throughout raise concerns of informed consent, which only rarely turn up in comparable non-biomedical interventions.

## Introduction

In the following I will try to vindicate the familiar thesis that biomedical enhancement raises moral concerns not raised by other means of human performance enhancement. A possible consequence from this thesis is: biomedical enhancement should be treated more carefully (e.g. with higher risk aversion) than other means of human performance enhancement. This is in a nutshell what Allen Buchanan has called and denounced as biomedical exceptionalism (Buchanan 2011b, 2011a).

It is not just Buchanan who rejects such an exceptionalism. Some of the most influential authors in the debate about human enhancement follow a similar – if not identical – path. Neil Levy bases his neuroethical thinking, including his thinking about neuroenhancement, on a moral parity thesis between biotechnological and more traditional interventions (Levy 2007, 2011),^1^ transhumanist writers explicitly refer to conventional means such as education and training and unconventional means, namely “nootropic drugs, gene therapy, or neural implants” (Bostrom and Sandberg 2009) and treat them more or less alike in moral terms. Some liberal writers such as John Harris accept that biomedical and non-biomedical improvements of human performance are to be treated alike morally (Harris 2007).

These authors can convincingly argue that the ethics of biomedical interventions can and should be treated without bias against a certain type of interventions and with the same rigor in identifying supportive arguments and objections as employed in other interventions. Most of them understandably argue against an anti-technological bias in bioethical thought. However, over-stressing the moral similarity of biomedical interventions with non-biomedical actions runs the risk of losing sight of the results of biomedical ethics and the complex set of ethical precautions which have been developed in this discipline. This risk lurks in the claim that biomedical enhancements require exactly the same moral considerations and precautions as non-biomedical enhancements.

In the following I will focus on Buchanan’s version of the claim that biomedical and non-biomedical enhancements require the same moral considerations, which he promoted together with in his influential and widely convincing discussion of human performance enhancement in general. Buchanan treats all methods of improving human performance as cases of enhancement for a good reason. He takes a human development perspective on enhancement and provides detailed analyses of the distributive effects of different enhancement interventions, be they biomedical or non-biomedical. For this purpose, different enhancements need to be morally comparable, and that can best be guaranteed by bracketing any special moral concerns associated with specific modes or means of enhancement. However, these brackets have to be eliminated once the moral analysis from the development perspective is established. There is a difference between bracketing and denying special moral issues associated with biomedical interventions.

### Buchanan on biotechnological exceptionalism

Buchanan has provided multiple formulations of biomedical exceptionalism. The strongest form reads like this:

“Biomedical enhancement exceptionalism—the dogmatic assumption that because an enhancement involves biotechnologies (pills, computers, fiddling with embryos, etc.) it’s somehow off the moral scale, that our ordinary moral tool kit is useless for coping with it.” (Buchanan 2011a)

Clearly, this form of biomedical exceptionalism will find few supporters within professional ethics, simply because it denies the competence of ethical thinking for the issues involved. That is, what it would mean that “our ordinary moral tool kit is useless for coping with it”, insofar as ethics is (or at least is a part of) our ordinary moral toolkit. There is, however, a more modest and more useful definition of biomedical exceptionalism:

“Second, I’ve shown that we should be wary of biomedical enhancement exceptionalism—of unthinkingly assuming that because an enhancement involves biomedical means, it must somehow be especially profound in its effects or especially morally problematic.” (Buchanan 2011a)

If we abstract from the rhetoric flavor introduced by the term “unthinkingly”, and the speculation about profoundness (whatever that is) this can stand as an initial working definition. And in this version, biomedical exceptionalism can, and does find proponents in professional ethics.

Def.: Biomedical exceptionalism := assuming that because an enhancement involves biomedical means, it must somehow be especially morally problematic.

There is a lot to unpack in this definition. First of all, it implies that ‘enhancement’ does not mean ‘biomedical intervention to improve human performance or welfare (beyond health)’, but rather is more inclusive. The term as used by Buchanan clearly can – is even intended to – refer to non-biomedical improvements of human performance. Buchanan makes this claim explicit in Better than human (Buchanan 2011a) and in Beyond humanity (Buchanan 2011b). ‘Enhancement’ is not to refer to biomedical interventions only. Some of his main examples such as numeracy, literacy, and agriculture refer to group-level cultural achievements, others seem to refer to individual tools such as glasses, microscopes etc. Second, this definition is intended to be comparative. Biomedical enhancement is thought to be more morally problematic than non-biomedical enhancement. Even if the latter were fully non-problematic, the biomedical version would still be problematic. Third, ‘morally problematic’ is a highly ambiguous term. It can either mean that something should be put forth as a topic of moral thought, or it can mean that something is to be considered vicious, impermissible, or has bad consequences. I will focus on the former, less ambitious claim here.

The case against biomedical exceptionalism had already been made in Buchanan’s slightly earlier *Beyond humanity*, where he insists that there isn’t “any reason to think that biomedical enhancements so defined are as such any more morally problematic than enhancements of other sorts. The *means* by which we pursue enhancements may, of course, matter morally; for example, enhancements that are imposed on those who do not wish to have them would be wrong. But that is not to say that the biomedical *mode* of enhancement is in itself distinctively problematic.” (Buchanan 2011b) The latter distinction is quite surprising, because voluntary vs. involuntary enhancement seems to fall under ‘mode’ of action much more naturally than under ‘means’. What is more, there is, as mentioned above, a number of authors who with good reason doubt that the *means* of interventions, i.e. the specific technology or technique in use, make much of a moral difference. The mode of action, however, is a common target of special bioethical deliberation. The biomedical mode seems to include some kind of practitioner-patient relationship, interventions based on a certain type of expert knowledge (Krieger 2011), with a certain set of goals (managing disease or prevention, diagnosis therapy, palliation), etc. Nevertheless, Buchanan ‘s claim for the time being seems to be that the *biomedical mode* of action does not raise any special moral concerns.

Given his working definition, what is Buchanan’s argument against biomedical exceptionalism? The argument for this claim is to be found in *Better than human*. His argumentative strategy is a mixture of shifting the burden of proof and refuting possible arguments of his opponents. The core claim which shifts the burden of proof is a form of parity assumption, namely that without any convincing arguments, biomedical interventions that improve human performance or abilities should be treated as morally on par with other means of such improvement.

Starting with this ethical parity assumption and the resulting distribution of the burden of proof Buchanan tries to identify what might speak for biomedical exceptionalism and refute each of the arguments he finds. His strategy of finding arguments for biomedical exceptionalism is to identify possible marks of distinction between biomedical and non-biomedical enhancements. Here are his candidates:

“(1) biomedical enhancements are different because they change our biology; (2) biomedical enhancements are different because (some of them) change the human gene pool; (3) biomedical enhancements are different because they could change or destroy human nature; (4) biomedical enhancements are different because they amount to playing God.” (Buchanan 2011a)

Buchanan’s arguments against the differences made in 2, 3 and 4 are more than convincing.^2^ The first difference, however, needs a little more scrutiny. The thesis that enhancements are different i.e. more morally problematic if they change human biology can be interpreted as referring to individual human biology and to shared biological traits. Consequently, Buchanan tries to refute both versions.

Buchanan does so by providing a number of counterexamples. First, he discusses one change in individual biology, which he seems to find clearly unproblematic: drinking coffee. Given that drinking coffee is morally unproblematic, he can point out a form of enhancement that changes human biology – much like a biomedical intervention – which is clearly not more morally problematic than non-biomedical enhancements such as literacy or numeracy, a false positive.

The second counterexample he provides is an involuntary change in biology, but not on a specific individual but in the population as a whole. Humans lost the ability to biosynthesize “vitamin C because of a random mutation that occurred in our lineage about forty million years ago.” (Buchanan 2011a) This alone merely shows that there are changes in human biology via evolutionary processes that can be considerably worse than those brought about by biomedical intervention. To complete the example Buchanan speculates about the morality of reversing this mutation in humans by genetic engineering. While he does not give a clear verdict, this thought experiment again seems to show that there are possible enhancements changing human biology which are morally unproblematic. The fact that Buchanan is more careful than with the coffee example likely owes to the fact that there are several authors who will object that such a genetic manipulation has its own moral problems.

His third counterexample is literacy, of which we know that it changes the structure of the human brain profoundly by reusing capacities which are used for other purposes (face-perception (Anderson 2014) in non-literate humans. This example is used to show how morally unproblematic changes in biology have enduring effect, while some biomedical enhancements have merely transient effects.

What these counterexamples show is: First, that there are morally accepted and acceptable transient and enduring changes of individual and species wide human biology (voluntary caffeine self-administration, literacy, possibly regaining vitamin-c biosynthesis). Second, there have been disadvantageous evolutionary changes in human biology (loss of vitamin-C biosynthesis) These results are, however, not sufficient to show that biomedical and non-biomedical forms of enhancing human performance or capacities are morally on par, neither in general nor in specific cases.

What Buchanan would have to show to refute biomedical exceptionalism is that in all cases in which biomedical and non-biomedical human performance enhancements have nearly equal aims and nearly equal risk–benefit-ratios there are exactly the same moral issues, i.e. no additional moral issue to be concerned with for either of them. Only then would he have refuted biomedical exceptionalism as the claim that biomedical enhancements are morally more problematic than non-biomedical ones. Because the universal quantifier makes this task impossible, it would suffice to show this for a relevant number of cases from different areas of human performance enhancement and with different means. His theoretical opponent, however, needs to show for one such situation that the biomedical enhancement carries additional moral issues. Because one singular case might be an exception to an otherwise good rule, he should provide a small number of such examples.

If results supporting Buchanan’s claim were to be forthcoming, it would really upturn the contemporary ethical landscape, simply because it would threaten to eradicate a major part of a sub-discipline of ethics, namely of biomedical ethics. Just one additional premise in needed to infer a radical conclusion, and one that has found quite a bit of support in the current literature: the premise that there is no principled – and no moral – distinction between therapy and enhancement. If therapy and enhancement, biomedical intervention and any other intervention are morally fully on par, there is much less need for biomedical ethics. While admittedly some of the main topics such as abortion and euthanasia would be unaffected, all the debates about moral issues specific to therapeutic interventions would become vacuous. Such a result would be surprising, admittedly it would radically increase theoretical elegance and austerity of ethical theory. But is it likely?

### The moral concerns of biomedical ethics

The case against biomedical exceptionalism has been supported with a number of example cases directly comparing biomedical and non-biomedical enhancement for the same aim. Neill Levy, following a contribution by Erik Parens, discusses the reduction of classroom sizes and the administration of Ritalin with the aim of improving concentration and thus educational success (Levy 2007). I want to argue that even this prototypical example does not help to make a case against, but rather for biomedical exceptionalism.

The case can be made vivid and at the same time advanced theoretically by engaging in a thought experiment:

Imagine the parents of one of the children in Parens and Levy’s example. One day they receive a letter from the head of the school. Now, distinguish two cases: In case A the letter informs them that the school will reduce classroom sizes, and from now on the child will have 19 instead of 29 classmates. The rest of the letter explains how the school hopes to improve pupil’s concentration and thus educational success, it details the costs of this change incurred by additional requirements for material and staff. In case B the letter informs them that the school will from now on professionally administer a dose of Ritalin*^3^ to all pupils early in the morning. The rest of the letter is identical, explaining expected effects and costs.

Here is an empirical claim: the moral reaction to both letters would be quite different. If you as the reader disagree and think that the parent would (and should) have the same reaction and moral attitude to both letters, the following argument will likely look futile for you. Please read on only for the sake of entertainment.

Admittedly, a part of the difference in reaction to the letter in case A and B merely reflects our moral and legal belief system as it contingently stands today. Reducing class sizes is an established, familiar technique while Ritalin* is unfamiliar and the widespread use for this purpose is not established. This thought experiment can however be refined in order to expose a difference that seems to go beyond mere contingent morality. The case with the second letter needs to be varied slightly for that purpose. We can imagine a set of slightly varied cases with this letter coming in several different versions B_1-n_. In these versions the cost of the intervention, including adverse effects, ranges from the amount given in the letter of case A to zero. The expected positive effect of the intervention is kept fixed at a level equal to that of case A.^4^ The question now is: Will there be a transition point, at which the parent reacts with the same *moral attitude* to the letter in case A and one of the letters in case B?^5^ Please mind: the variable of interest is the attitude i.e. the set of moral concerns the person has concerning the intervention in question, not the final verdict. It might still be the case that someone receiving this letter has additional moral concerns, would wish for additional precautions or an option to withdraw, i.e. have a different moral attitude, but is persuaded by cost–benefit considerations and thus comes to the same final verdict.

Agreeing that there is such a transition point implies accepting that there are no additional moral concerns in case B compared to A. The theoretical costs of such an agreement are, however, high. Amongst others, this implies that one would have the same concern regarding requirements for consent: If the child is not involved into the decision whether to reduce classroom size, neither is it asked for assent to taking Ritalin*. The same goes for the parent: they are being informed that these measures are being taken, not asked for consent. The exit options are the same for the two cases: one can only take the child to a different school, not refuse to take the pill or to stick to a larger class.

If, on the other hand, one considers the extra concerns of – amongst others – informed consent to be justified in biomedical cases but would not agree that they are raised by non-biomedical forms of enhancement such as reducing class size, there seems to be a case for biomedical exceptionalism.

Several equivalent examples can easily be created: In many contexts, ranging from school to the workplace or even the political arena, we accept some interventions into our surrounding and routines without the explicit need for consent, much less informed consent. If the same effects were to be generated by biomedical interventions, we require some provision for consent or at least assent. That we do, seems to provide the counterexamples to Buchanan’s ethical parity thesis which we were looking for. Thus, it seems to make a case for biomedical exceptionalism. There are however a number of possible objections.

## Objections

The first objection against the case for biomedical exceptionalism attacks the argumentative strategy which led to the current result. It claims that biomedical exceptionalism has an even higher burden of proof than suggested above.

The argument above does re-burden the proponent of the ethical parity thesis with showing that medical and non-medical interventions with the same risk–benefit ratio give rise to the same moral concerns. The exceptionalist claims that he can shift the burden of proof, because he has shown some *prima facie* examples of medical and non-medical interventions raising *different* moral concerns. He or she thinks that it now is on his opponent to show how these *prima facie* different moral concerns are not different after all. But that might be seen as too rash a redistribution of the burden of proof. According to this objection the biomedical exceptionalist first has to show that *no* non-biotechnological intervention raises the concerns which have been identified for biomedical interventions.

On the one hand, this concedes slightly more than Buchanan’s original burden-of-proof-argument, as introduced above. Buchanan insists that the biomedical exceptionalist needs to demonstrate that there are special moral concerns for biomedical interventions in the first place. The objection just introduced would accept the existence of such concerns. On the other hand, this objection lays even stronger demands on the biomedical exceptionalist. It casts doubt on whether these concerns are specific and exclusive for biomedical interventions. Thus, it is methodologically extremely demanding because it requires the exceptionalist to support a negative existential claim: there are no non-biotechnological interventions that raise concerns also raised by biotechnological ones.

In order to show how this extremely demanding requirement is reasonable, the opponent of biomedical exceptionalism can raise the possibility that interventions do not raise the concerns mentioned above because they are biomedical, but because of some other, more inclusive property. The concerns typically associated with biomedical interventions are, according to this objection, raised by a larger set of actions, of which biomedical interventions just happen to be a subset. Such a larger set might either be based on some further similarity of the actions in question.^6^ The opponent of biomedical exceptionalism could for instance suggest that all actions directly affecting another person’s body raise similar concerns. This set includes a subset of medical interventions and a subset of non-medical interventions such as a haircut.

This set will, however, not support the case against biomedical exceptionalism. It is too unclear what ‘directly affecting’ is supposed to mean. Invasiveness (breaking the skin barrier), touch, transfer of energy might be the first criteria most people will think of, but sensory stimulation of all kinds is a similarly good candidate. There are actions that in this sense directly affect another person’s body but which seem to raise no moral concern whatsoever – catching someone’s fall, – and others which barely affect others bodily *directly*, but still raise significant moral concerns such as psychotherapy or even sexual orientation change efforts, sugarcoated as ‘conversion therapy’.

In general, this relocation of the burden of proof can only work if there is a good candidate for a larger set of actions, which comprises biomedical interventions and several others, and which gives rise to the concerns typically associated with biomedical interventions. It might be due to a lack of imagination on my side, but at the moment no such candidate seems to be present.

The second, related objection targets the details of the argumentative strategy above. It raises doubts whether the examples I provided in support of biomedical exceptionalism might merely pick out two rather random points is the distribution of practices that raise different moral concerns. Other biomedical example might raise less, other non-biomedical examples more moral concerns. To support this case against biomedical exceptionalism, one might provide several examples for non-biomedical interventions which justify moral concerns typically associated with biomedical procedures. If it turns out that most, or even all, the common concerns connected to biomedical interventions are also raised by some non-biomedical interventions, this might be taken as evidence against biomedical exceptionalism. My example in the letters A and B_1-n_ would then have been overly suggestive. Imagine for example, that in a case C the letter informs the parent that the school is going to install a well-established set of didactic methods from military boot camps. Strict obedience, harsh punishments, continuous surveillance, stark limitation to expressions of individuality in language, clothing, hairstyle and behavior. Again, the rest of the letter outlines exactly the same risk–benefit considerations as above. Wouldn ‘t the parents’ reaction in case A and C be very different indeed, and would not the parent have different moral concerns in these two situations?

It should indeed be conceded that different interventions – biomedical as well as non-biomedical – raise different sets of concerns and require different sets of precautions. In the case of the boot camp style methods, parents might have concerns regarding the free development of their child’s personality, its freedom of expression etc., and they would – if they do not immediately exit – surely request extensive monitoring requirements, compensatory activities outside school, counselling opportunities etc. But that alone does not suffice to refute the argument above. That different non-biomedical interventions raise different moral concerns does not mean that any of them, much less most of them, raise the same concerns as biomedical interventions.

Nevertheless, this objection helps to point out something important about biomedical exceptionalism. Following the working definition above, biomedical interventions are especially morally problematic. This should not be taken to mean that there are no non-biomedical interventions which raise more and more grievous moral concerns than some biomedical intervention. It should, however, be taken to mean that by falling into the domain of biomedical action, an intervention raises additional moral concerns, which would not be raised by a comparable intervention which does not employ biomedical means. Understood like this, biomedical exceptionalism would either be refuted if biomedical interventions did not raise any special concerns, or if their raising such concerns were not due to their character as *biomedical* interventions. The first refutation attempt has hopefully been rebutted by the argument in the first part of this article. The second has been raised and rejected in the previous objection. There is, however, a variation of this refutation attempt, which gives rise to a third objection.

The third objection again attacks details of the argument above. It elaborates on the doubt already present in the example, whether the different reactions to the two letters really owes to and is justified by the difference between biomedical and non-biomedical interventions. The differences in reaction can have several causes, e.g. that in one case there is a familiar technique, in the other an unfamiliar technological one, or that in one case the intervention in question is a part of our life world while in the other it is not. Thus, our moral reactions wouldn’t track the difference *biomedical vs non-biomedical* but differences such as *familiar versus unfamiliar*, or *social versus technological*. One could claim – and Buchanan seems to do so in a number of passages – that the difference in our reactions to biomedical and non-biomedical interventions – is a case of mere conservatism.

What is more, even the justification for raising the most prominent biomedical concern, namely concern of consent and insisting on informed consent-procedures in one and not the other case, one might claim, has nothing to do with one intervention being biomedical. Common bioethical practice – if not theory – shows that the real difference lies between high and low risk procedures. In daily life we do not insist on a formal consent procedure when a doctor collects a sputum sample nor even when he takes a blood sample. We do not, because these are common, very low risk interventions. We do insist on a consent procedure for surgical interventions and the like because the risk is higher. If indeed differences in moral reaction tracked familiarity or risk profile, it would indeed not make much of a difference for our moral evaluation whether an enhancing intervention is biomedical or not. The real difference would be between different perceived risk profiles.

This objection runs into a dilemma, however. Either it relies on common moral practice. If it does, it must take into account that biomedical interventions are indeed enmeshed in a much stricter web of moral debates and precautions than other areas of conduct. Pointing out one difference within this practice alone – the difference of enforcement of consent procedures in higher or lower risk interventions – is cherry picking evidence. Or it relies on the local ethical theories for biomedical and other practices. Then it will have to take into account that informed consent is a requirement for all biomedical interventions, familiar or not, technological or not, because a patient needs to suspend his right to bodily integrity for a doctor to be justified in performing the intervention. A consent requirement admittedly does exist beyond biomedical practice. Mere consent requirements – as opposed to *informed* consent – are quite common, ranging from contract law to sexual relationships. In the specific form of *informed* consent, it is established in the large but structurally similar area of research with human participants (expert-layperson relationship, similar involvement of expert knowledge, suspension of rights by the participant). Furthermore, the information requirements for informed consent procedures vary within types of research with biomedical research – i.e. including biomedical interventions – being most similar to the one of *medical* intervention. That it is *biomedical* research, which is embedded within the most similar set of precautions and raises the most similar concerns seems to support biomedical exceptionalism rather than cast doubt on it.

Another objection against my argumentative strategy targets the distinction between moral concerns and moral verdict. It claims that there will always be a transition point at which people will change their attitude towards an intervention. That there always is such a transition point is a basic assumption of decision theory. As research on absolute values^7^ shows, there are situations in which these absolute values need to be and indeed are balanced against non- absolute values (Fiske and Tetlock 1997). And since such decisions are actually being made, they are possible, meaning that values are in general commensurable, even if the decision process is extremely discomforting for the subject (Hanselmann and Tanner 2008). Following this line of thought, the different attitudes do not show that there are any concerns specific to biomedical interventions, but only that we have invented specific precautions in the case of biomedical interventions, which might be efficient ways of safeguarding what we value, but which are in principle interchangeable much as the values themselves are commensurable with risk and benefit.

This objection relies on presuming what it intends to show. It presumes that in forced decisions of the tragical (absolute vs absolute values) or the taboo (absolute vs. non- absolute value) type there is commensurability. But from the fact that a decision needs to be taken, nothing follows about the commensurability of values. Assuming that by fiat of a decision in tragic situations values become commensurable simply ignores that these situations are tragic and that the values in question cannot be weighed against each other.

### A conciliatory objection: the case against bio*technological* exceptionalism

In some passages Buchanan seems to argue that he is much less concerned with the difference between biomedical and non-biomedical interventions but much more with biotechnological versus non-biotechnological interventions. And indeed, biomedical actions including non-technological interventions such as massage, physiotherapy, etc. do raise the same concerns as technologically supported ones such as pharmaceuticals, surgeries or implants. It does not seem to play a significant role, whether the intervention into a person’s body happens via some technological path or via some purely manual means. The person still needs to suspend her law to bodily integrity, still needs to be informed about the details and consequences of the intervention, etc.

It would be a different but much better supported argument if it were one against bio*technological* exceptionalism and rejected the claim that that because an enhancement involves bio*technological* means, it must somehow be especially morally problematic. This is at odds with Buchanan’s claim from above: “The *means* by which we pursue enhancements may, of course, matter morally; for example, enhancements that are imposed on those who do not wish to have them would be wrong. But that is not to say that the biomedical *mode* of enhancement is in itself distinctively problematic.” (Buchanan 2011b) Contrary to this passage, many of Buchanan’s arguments can be read as a form of ‘technological veil of ignorance’, the most plausible suggestion to look at the real risks and benefits of an intervention and not at the type of technology involved. Thus, if Buchanan referred with ‚biomedical mode ‘ to *bio*medical interventions, i.e. the use of intervention based on the biosciences, as opposed to bio*medical interventions*, i.e. the use of expert knowledge for the sake of managing disease in a practitioner-patient relationship, his argument against *bio*medical exceptionalism would be more convincing. It would moreover be better aligned with the parity theses of Levy, Harris and others. However, it just happens to be the case that most and especially the most striking *bio*medical (and biotechnological) interventions – therapeutic and performance enhancing – are bio*medical* interventions and thus still raise all the concerns and issues discussed in biomedical ethics.

## Footnotes

1 For a detailed discussion of this ethical parity thesis see for example (Heinrichs 2017).

2 Buchanan refutes arguments based on human nature as well as the idea of change to the human gene pool in (Buchanan 2009). Both the reference to human nature and to playing god can be seen as placeholder debates obscuring the underlying moral issues as discussed in (Rüther and Heinrichs 2019).

3 Ritalin seems not to have the effect on healthy subjects which users and proponents of enhancement claim and hope it does (Repantis et al. 2010). For the sake of the thought experiment I’ll refer to Ritalin* as a more potent enhancement-drug.

4 Please mind that this article does not claim that the effectiveness and side-effects of Ritalin and decreased of classroom sizes are the same. The scenario is completely hypothetical. While there is – and given the provisions for research with human participants – will be no direct empirical comparison, research into the substance and into the effect of classroom size reduction indicate that the effects and side effects are quite distinct, much to the advantage of the latter.

5 This is basically what Levy claims in his discussion of the example: “it cannot be because Ritalin allows us to achieve, at a lower cost, *precisely the same* results (in terms of academic performance and well-adjusted students) as better classrooms and more individualized teaching. If it does, then Ritalin is clearly *superior*: if it has the same benefits, and lower costs, then we should use it (and use the savings elsewhere, where the money is needed).“ (Levy 2007).

6 They might, even worse, be held together just by the similarity of the concerns they raise. I’ll return to the latter possibility later on, as it raises doubts whether my example for special moral concerns about biotechnology show anything at all.

7 The research tradition in question tends to use the term “sacred” value. However, the values in question are characterized by their importance and validity, not by some religious belief system into which they are embedded. That is why the term ‘absolute’ will be used in this article.
